# Distinct Cytokine and Chemokine Profiles in Autism Spectrum Disorders

**DOI:** 10.3389/fimmu.2017.00011

**Published:** 2017-01-23

**Authors:** Yvonne M. Y. Han, Winnie K. Y. Cheung, Chun Kwok Wong, Sophia L. Sze, Timmy W. S. Cheng, Michael K. Yeung, Agnes S. Chan

**Affiliations:** ^1^Department of Rehabilitation Sciences, The Hong Kong Polytechnic University, Hong Kong, China; ^2^Neuropsychology Laboratory, Department of Psychology, The Chinese University of Hong Kong, Hong Kong, China; ^3^Department of Chemical Pathology, Prince of Wales Hospital, The Chinese University of Hong Kong, Hong Kong, China; ^4^Chanwuyi Research Center for Neuropsychological Well-Being, The Chinese University of Hong Kong, Hong Kong, China

**Keywords:** immunologic function, autism, cognitive function, hyperactivity, comorbidity

## Abstract

Previous studies have shown that immunological factors are involved in the pathogenesis of autism spectrum disorders (ASDs). However, this research has been conducted almost exclusively in Western contexts, and only a handful of studies on immune measures have been conducted in Asian populations, such as Chinese populations. The present study examined whether immunological abnormalities are associated with cognitive deficits and problem behaviors in Chinese children with ASD and whether these children show different immunological profiles. Thirteen typically developing (TD) children and 22 children with ASD, aged 6–17 years, participated voluntarily in the study. Executive functions and short-term memory were measured using neuropsychological tests, and behavioral measures were assessed using parent ratings. The children were also assessed on immunological measures, specifically, the levels of cytokines and chemokines in the blood serum. Children with ASD showed greater deficits in cognitive functions, as well as altered levels of immunological measures, including CCL2, CCL5, and CXCL9 levels, compared to TD children, and the cognitive functions and associated behavioral deficits of children with ASD were significantly associated with different immunological measures. The children were further sub-classified into ASD with only autistic features (ASD-only) or ASD comorbid with attention deficit hyperactivity disorder (ASD + ADHD). The comorbidity results showed that there were no differences between the two groups of ASD children in any of the cognitive or behavioral measures. However, the results pertaining to immunological measures showed that the children with ASD-only and ASD + ADHD exhibited distinct cytokine and chemokine profiles and that abnormal immunologic function was associated with cognitive functions and inattention/hyperactivity symptoms. These results support the notion that altered immune functions may play a role in the selective cognitive and behavioral symptoms of ASD.

## Introduction

One essential finding in autism spectrum disorder (ASD) research has been the consistent immunological abnormality detected among autistic individuals. Although it is largely unknown how the immunologic factor specifically affects the neural networks in the brains of individuals with ASD, dysfunctional immune profiles involving inflammatory changes in the CNS have been documented in ASD. For example, increased levels of inflammatory cytokine transforming growth factor beta 1 [TGFβ1; (1)], chemokine macrophage chemoattractant CCL2 (1), and CXCL8 (2) were found in the brains of individuals with ASD. In addition to the elevated neuroinflammatory response in the autistic brain, altered levels of circulating cytokines and chemokine have also been reported. For example, higher levels of macrophage migration inhibitory factor (MIF) and CXCL8 were observed in plasma and cerebrospinal fluid (CSF) specimens from individuals with ASD compared with those from typically developing (TD) controls and subjects with other developmental disabilities (1, 3–5). In addition, elevated plasma CCL5 and CCL2 were observed in ASD, whereas increased CCL2 found newborn bloodspot specimens have been shown to be related to the risk of ASD (1, 6, 7). Similarly, altered levels of TGFβ1 have also been found in plasma and serum specimens of ASD compared with TD controls (8–10). These findings suggest that the increased inflammatory chemokine and cytokine production detected in the brain and peripheral blood of autistic individuals may produce a profoundly negative effect on proper neuronal development, migration, differentiation, and synapse formation and subsequently affect the behavior of individuals with ASD (1, 11, 12). Consistent with these findings, cytokine and chemokine levels have been shown to correlate with the severity of behavioral abnormalities in individuals with ASD. Increased plasma levels of MIF have been correlated with more severe social impairment and decreased imaginative play (4), whereas lower TGFβ1 levels have been associated with more stereotypy, irritability, hyperactivity, and other behavioral symptoms and fewer adaptive behaviors (8). Significant associations between increased plasma levels of CCL5 and CXCL8 and more frequent aberrant behaviors and fewer adaptive behaviors have also been found (3, 6).

Although the aforementioned studies have indicated plausible links between the severity of certain core behavioral symptoms and shifts in cytokine and chemokine levels in ASD, it is important to note that the exploration of neuroinflammatory mechanisms in ASD has been based predominantly on research conducted in Western populations, of which the expression of autistic traits does not necessarily extend to all populations. For example, ASD children from Western populations (United Kingdom and United States) showed significantly greater deficits in the domains of non-verbal communication/socialization and insistence on sameness/restricted interests than ASD children from Eastern populations (Israel and South Korea), as was demonstrated in a study conducted by Matson et al. (13). Therefore, it may be worth considering whether the immunological profiles in ASD differ according to ethnicity. Moreover, it will be interesting to determine whether the observed ethnic differences in the symptoms of ASD are reflected in their immunological profiles such that ethnic differences are also observed in the levels of cytokine and chemokine production among autistic individuals from different ethnic backgrounds. Hence, our present study attempted to add new knowledge regarding the ethnic aspects of immunological dysfunctions in the peripheral blood of Chinese individuals with ASD.

Furthermore, although the relationship between the levels of cytokine and chemokine production and the severity of cognitive outcomes have been detected in ASD (3, 4, 6, 8), reports of peripheral chemokine levels have been inconsistent. For example, while higher levels of CCL2, CCL5, and CXCL10 were found in individuals with ASD than in TD controls in either plasma or CSF specimens (1, 6, 7), lower plasma levels of these three chemokines were detected in ASD subjects with fragile X syndrome (FXS, a single-gene disorder characterized by autistic symptoms) compared to TD controls (14). Similarly, several studies have reported decreased levels of TGFβ1 measured in plasma and serum (8–10), whereas the opposite trend has been observed in the brain tissue of individuals with ASD (1). Considering the discrepancies in the levels of cytokine and chemokine production reported in previous studies, it is questionable whether this relationship arises simply because chemokine and cytokine levels vary between different samples or because of the complexity of the clinical diagnosis of ASD, in which autism may be comorbid with symptoms associated with other neurological disorders, leading to the expression of different immunological profiles. Indeed, differing levels of chemokines were found between individuals with ASD + FXS and individuals with ASD-only, as well as between autistic individuals with regression and those with early onset of ASD (3, 14). Because there is a significant proportion of individuals with ASD comorbid with AD/HD symptoms (ASD + ADHD) who demonstrate a more severe profile of cognitive and behavioral impairment compared to individuals with only autistic features, this study also aimed to explore whether individuals with ASD and individuals with ASD + ADHD demonstrate different immunological profiles in terms of cytokine and chemokine production levels (15–19).

The purpose of the present study was therefore to (a) explore whether the patterns of the serum cytokine levels and chemokine production observed in individuals with ASD from Western populations would be replicated in ASD children from Eastern populations and (b) investigate whether the immunological measures of ASD would be associated with the severity of their behavioral and cognitive symptoms. To further highlight the relationship between altered immune functions and the diversity of autistic phenotypes, this study also aimed to (c) determine whether individuals diagnosed with ASD and co-diagnosed with ASD and ADHD would demonstrate different cytokine and chemokine profiles compared to their age- and IQ-matched TD counterparts. Drawing together the pieces of evidence linking ASD with altered immunologic function, we hypothesized that relative to TD children, Chinese children with ASD would show altered levels of inflammatory chemokine and cytokine in the blood serum. In addition, given that altered pro-inflammatory profile might be associated with neuronal damage which leads to the behavioral abnormalities in individuals with ASD, it was hypothesized that the specific panels of pro-inflammatory cytokines and chemokines would be associated with the severity of the cognitive dysfunction and behavioral problems in the children with the disorder. Finally, considering the discrepancies in the levels of cytokine and chemokine production reported in previous studies, it was further hypothesized that the children with ASD would have a different cytokine and chemokine profile from those comorbid with ADHD.

## Materials and Methods

### Participants

Participants in the study were recruited by posting advertisement on our websites and sending emails to parents in our existing database at the Neuropsychology Laboratory of the Chinese University of Hong Kong. Seventeen TD children and 23 children with ASD, aged 6–17 years, participated voluntarily in the study with their parents’ written consent. All children with ASD were diagnosed by a clinical psychologist based on the diagnostic criteria of DSM-V (20), and the information collected from the Autism Diagnostic Interview-Revised [ADI-R; (21)]. Of the 23 children with ASD, 10 children also demonstrated prominent symptoms of attention deficit hyperactivity disorder (ADHD) that warranted a comorbid diagnosis of ASD and ADHD (ASD + ADHD) and 13 met the diagnostic criteria of ASD with no or only mild ADHD-like features that did not meet the diagnostic criteria of ADHD (ASD-only). The TD children had no history of delay in developmental milestones or any neurological or psychiatric disorders as reported by their parents; the children were screened for autism traits using the ADI-R and the Social Responsiveness Scale [SRS-2; (22)]. One ASD + ADHD child who was prescribed immunosuppressive drugs was excluded from the study, and four children from the TD group were also excluded because their scores were either above the cutoff scores of 10, 9, and 3 in the three subscales (reciprocal social interaction, communication, repetitive/restricted and stereotyped patterns of behaviors, respectively) of the ADI-R (*n* = 3), or of the total raw score of 70 in the SRS-2 (*n* = 1).

Tables 1 and 2 shows the demographic and clinical characteristics of the children. The ASD and TD groups were matched on age, *t* = 1.17, *p* = 0.26, and intellectual functioning, *t* = 1.80, *p* = 0.09, as measured by the short form of the Chinese version of the Wechsler Intelligence Scale for Children-Fourth Edition (Hong Kong) [WISC-IV (HK)] (23). The ASD-only group contained significantly more males than the other two groups, likelihood ratio = 6.49, *p* = 0.04. The TD group demonstrated significantly fewer ASD or ADHD related symptoms than either ASD group (*F* ranges from 30.56 to 51.42, *p* < 0.001). Although the level of behavioral problems between the two ASD groups did not reach statistical significance, the ASD-only group demonstrated slightly more ASD-related features than the ASD + ADHD group.

**Table 1:** **Demographic and clinical characteristics of children with ASD (*n* = 22) and TD controls (*n* = 13)**.

	TD (*n* = 13)	ASD (*n* = 22)	*t* or χ*^2^*	*p*
Age, years	10.92 (3.95)	9.50 (2.54)	1.17	0.26
Gender—male (%)^a^	69.23	90.91	2.62	0.11
IQ	106.69 (13.23)	99.36 (8.29)	1.80	0.09
ADI-R social interaction	3.85 (2.91)	18.55 (6.77)	8.89	<0.001**
ADI-R communication	1.31 (1.38)	13.64 (5.27)	10.39	<0.001**
ADI-R stereotyped behavior	0.23 (0.60)	5.18 (2.24)	9.80	<0.001**
SRS-2 total score	28.77 (10.29)	78.55 (25.94)	8.00	<0.001**

*Data are presented as means (SD)*.

*ADI-R, Autism Diagnostic Interview-Revised; IQ, intelligence quotient as assessed by the Chinese version of Wechsler Intelligence Scale for Children Forth Edition; SRS-2, Social Responsiveness Scale Second Edition; ASD, children with diagnosis of autism spectrum disorder; ASD-only, children with diagnosis of autism spectrum disorder only; ASD + ADHD, children with comorbid diagnosis of autism spectrum disorder and attention deficit hyperactivity disorder; TD, typically developing children*.

*^a^Likelihood ratio Chi-square test were performed for distribution violating the sample size assumption of Chi-square test*.

**p < 0.01.

**Table 2:** **Demographic and clinical characteristics of children with ASD only (*n* = 13), children with comorbid diagnosis of ASDs and attention deficit hyperactivity disorder (*n* = 9), and TD controls (*n* = 13)**.

	TD (*n* = 13)	ASD		*F* or χ*^2^*	*p*	*Post hoc* results
		ASD-only (*n* = 13)	ASD + ADHD (*n* = 9)			
Age, years	10.92 (3.95)	9.38 (2.84)	9.67 (2.18)	0.84	0.44	
Gender—male (%)^a^	69.23	100.00	77.78	6.49	0.04*	
IQ	106.69 (13.23)	100.08 (9.30)	98.33 (6.98)	2.06	0.14	
ADI-R social interaction^b^	3.85 (2.91)	19.31 (7.18)	17.44 (6.37)	37.20	<0.001**	TD < ASD-only, ASD + ADHD
ADI-R communication^b^	1.31 (1.38)	13.77 (4.90)	13.44 (6.06)	51.42	<0.001**	TD < ASD-only, ASD + ADHD
ADI-R stereotyped behavior^b^	0.23 (0.60)	5.85 (2.19)	4.22 (2.05)	50.51	<0.001**	TD < ASD-only, ASD + ADHD
SRS-2 total score^b^	28.77 (10.29)	81.08 (28.68)	74.89 (22.51)	30.56	<0.001**	TD < ASD-only, ASD + ADHD

*Data are presented as means (SD)*.

*ADI-R, Autism Diagnostic Interview-Revised; IQ, intelligence quotient as assessed by the Chinese version of Wechsler Intelligence Scale for children Forth Edition; SRS-2, Social Responsiveness Scale Second Edition; ASD, children with diagnosis of autism spectrum disorder; ASD-only, children with diagnosis of autism spectrum disorder only; ASD + ADHD, children with comorbid diagnosis of autism spectrum disorder and attention deficit hyperactivity disorder; TD, typically developing children*.

*^a^Likelihood ratio Chi-square test were performed for distribution violating the sample size assumption of Chi-square test*.

*^b^Welch’s test and Games–Howell post hoc test were performed for variables violating the homogeneity of variances assumption of ANOVA test*.

*p < 0.05, **p < 0.01.

### Procedure

This study was conducted in accordance with the Helsinki Declaration of the World Medical Association Assembly. The research protocol was approved by the Joint Chinese University of Hong Kong—New Territories East Cluster Clinical Research Ethics Committee (Ref. No. 2013.520). Prior to the assessment, all of the children and their parents were briefed on the procedure of the study, and informed consent was obtained from the parents. Peripheral blood samples and data on neuropsychological and behavioral measures were collected on separate days.

A registered nurse drew 3 ml of EDTA blood and 3 ml of clotted blood from each child using venipuncture at a medical clinic. Each blood sample was centrifuged at 3,000 rpm for 15 min, and the harvested serum was then stored at −80°C in a clinical laboratory until cytokine and chemokine levels were measured. Serum cytokine and chemokine concentrations were measured following the manufacturer’s instructions. Blood sample processing and assay were performed by an experienced laboratory technician who was blind to the clinical characteristics and group assignments of the participants.

Neuropsychological assessments of the children and clinical interviews of the parents were administered on another day by well-trained research assistants. The neuropsychological assessment involved standardized tests on two cognitive domains (executive functioning and short-term memory) that are frequently found to be problematic in individuals with ASD (24, 25). In the clinical interview, information about the children’s developmental and medical history, as well as past and present socio-emotional and behavioral characteristics, was collected from the parents through a structured interview and standardized questionnaires. The diagnosis of each child was confirmed by a clinical psychologist based on the DSM-V criteria and the ADI-R. The research assistants who conducted the assessment, the nurse who drew the blood, and the technician who performed the blood assays were blinded to the rationale of the study and the group assignment.

### Measures

#### Immunological Measures

The concentrations of the cytokines TGFβ1 and MIF were measured using an enzyme-linked immunosorbent assay (R&D Systems, Inc., MN, USA) method, whereas those of the chemokines CCL2, CCL5, CXCL8, CXCL9, and CXCL10 were assessed using BD™ human chemokine cytometric bead array (CBA) reagent (Becton Dickinson Biosciences Pharmingen, CA, USA). Samples were analyzed on a multi-fluorescence BD FACSCalibur™ flow cytometer using BD CellQuest™ and BD™ CBA software.

#### Cognitive Measures

##### Executive Functioning

Four well-defined measures of executive functioning in planning and organization, cognitive flexibility, and generativity were used in the present study: (1) the Tower of London Test-Drexel Version (TOL^DX^) (26), (2) completion time of the second trial of the Children’s Color Trail Test [CCTT; (27)], (3) the total number of unique designs of the Five Point Test [FPT; (28)], and (4) the Copy trial of the Rey–Osterrieth Complex Figure Test [Rey-O; (29)]. The TOL^DX^ requires movement of three colored beads on three vertical pegs to match a target arrangement while adhering to the test rules. The second trial of the CCTT requires connecting scattered numbers in ascending order and concurrently alternating between pink and yellow. The FPT requires the production of novel designs by connecting five points with straight lines within 5 min. The Rey-O Copy task involves copying a complex geometric figure and requires sufficient attention and concentration and the ability to organize the figure into a perceptual whole.

##### Short-term Memory

Short-term memory comprises two measures tapping the ability to temporarily store new information in memory: (1) total scores on Digit Span—Forward and Backward (DS) of the WISC-IV (HK) (23) and (2) the immediate recall trial of Rey-O. The Rey-O task requires drawing a complex figure from memory after copying the figure, and the DS requires the repetition of sequences of random numerals read aloud by the examiner.

#### Behavioral Measures

##### Social Communication/Interaction

This measure consists of three measures derived from two standardized questionnaires tapping core deficits in social functioning of ASD: (1) reciprocal social interaction, (2) communication subscale scores of the ADI-R, and (3) social communication/interaction total scores on the Social Responsiveness Scale, Second Edition [SRS-2; (22)].

##### Repetitive/Restricted Behavior

This measure consists of two behavioral measures tapping another set of core ASD symptoms in the repetitive, restricted behavior repertoire, interests or activities: (1) restricted, repetitive, and stereotyped patterns of behaviors subscale scores on the ADI-R and (2) repetitive/restricted behaviors subscale scores on the SRS-2.

##### Inattention/Hyperactivity

This measure involves three measures derived from standardized questionnaires tapping core AD/HD symptoms of inattention and hyperactivity/impulsivity: (1) cognitive problems/inattention, (2) hyperactivity, and (3) ADHD index subscale scores on the short version of Conners’ Rating Scales-Revised [CRS-R; (30)].

### Data Analyses

Cognitive functions and behavioral measures were compared and examined for differences between the ASD and TD groups. To reduce the number of statistical comparisons, two composite scores of the cognitive domain (*executive functioning, short-term memory*) and three composite scores of the behavioral domain (*social communication/interaction, repetitive/restricted behavior, inattention/hyperactivity*) were computed by summing and averaging the *Z* scores from the corresponding cognitive and behavioral measures of the different cognitive and behavioral domains. Low scores indicated poor performance in cognitive functioning and impaired behavior. To examine differences between the ASD and TD groups of children, the two cognitive composite and the three behavioral composite scores were compared using the independent-samples *t*-test (independent *t*-test). For the immunological measures, the concentrations of the cytokines and chemokines were compared between the ASD and TD groups using independent *t*-tests.

To determine whether ASD and ASD + ADHD would demonstrate different cytokine and chemokine profiles compared to their TD counterparts, between-group comparisons on continuous variables were performed using multivariate analysis of variance (MANOVA) followed by separate univariate analysis of variance (ANOVA) and *post hoc* Tukey HSD tests. Between-group comparisons on categorical variables were analyzed using Chi-square tests. For distributions that had more than 20% of cells with an expected count of less than 5, the likelihood ratio was reported. Spearman’s correlation was performed to analyze the association between two continuous variables. All statistical analyses were performed using the SPSS software (SPSS, Inc., Chicago, IL, USA). Given that specific hypotheses were tested, the alpha level was not adjusted to avoid reducing the power of the tests, and the corresponding effect sizes of different statistics were reported to determine the strength of association and degree of difference based on Cohen (31) and Hopkins (32) criteria.

## Results

### Comparison of Cognitive and Behavioral Measurements between Children with ASD and TD Controls

Children with ASD showed significantly lower composite scores in all cognitive domains: executive functioning, *t*(33) = 2.09, *p* = 0.04, and short-term memory, *t*(33) = 2.90, *p* = 0.007; and in all behavioral domains: social communication/interaction, *t*(33) = 11.93, *p* < 0.001, repetitive/restricted behavior, *t*(33) = 10.23, *p* < 0.001, and inattention/hyperactivity, *t*(33) = 6.01, *p* < 0.001, compared to TD controls, indicating deficient cognitive and behavioral functioning in ASD (Table 3).

**Table 3:** **Comparison of mean composite scores in cognitive and behavioral domains in children with ASD (*n* = 22) and TD controls (*n* = 13)**.

	TD (*n* = 13)	ASD (*n* = 22)	*t*	*p*
Executive functioning	−0.30 (0.50)	−0.71 (0.60)	2.09	0.04*
Short-term memory	−0.08 (0.70)	−0.74 (0.62)	2.90	0.007**
Social communication/interaction	3.10 (0.37)	−0.19 (1.20)	11.93	<0.001**
Repetitive/restricted behavior	1.53 (0.21)	−0.88 (1.07)	10.23	<0.001**
Inattention/hyperactivity	0.22 (0.51)	−1.48 (1.14)	6.01	<0.001**

*Data are presented as means (SD)*.

*ASD, children with diagnosis of autism spectrum disorder; ASD-only, children with diagnosis of autism spectrum disorder only; ASD + ADHD, children with comorbid diagnosis of autism spectrum disorder and attention deficit hyperactivity disorder; TD, typically developing children*.

*p < 0.05, **p < 0.01.

### Comparison of Immunological Profiles between Children with ASD and TD Controls

Concentrations of CCL2 and CCL5 were significantly higher in children with ASD than in those with TD [CCL2: *t*(33) = −2.27, *p* = 0.03; CCL5: *t*(33) = −3.07, *p* = 0.004] (Table 4). Moreover, concentration of CXCL9 was approximately twofold lower in ASD children than in TD children, *t*(33) = 4.02, *p* = 0.001. No significant differences in the concentrations of MIF, TGFβ1, CXCL8, and CXCL10 were observed in children with ASD compared with those observed for TD children, *p* > 0.05. The results were consistent when only male participants in each group were compared. That is, significant between-group differences were found in CCL2, *t*(27) = −1.93, *p* = 0.03, CCL5, *t*(27) = −2.28, *p* = 0.03, and CXCL9, *t*(27) = 3.69, *p* = 0.02, suggesting that a gender difference was not a confounding factor that could account for differences in the immunological measures between the ASD and TD groups of children. Similarly, analysis of covariance was conducted to examine whether the differences between the TD and ASD groups could be attributed to age differences. Similar patterns of statistical significance were observed between the two groups after age adjustments were made, in which the level of CCL5 was significantly higher, *F*(1, 32) = 5.33, *p* = 0.03, and the CXCL9 level was significantly lower, *F*(1, 32) = 23.09, *p* < 0.001, in children with ASD than their TD counterparts. Nevertheless, although the level of CCL2 showed a higher trend in the ASD group after an adjustment was made for age, the difference between the ASD and TD groups did not reach statistical significance, *F*(1, 32) = 3.23, *p* = 0.08.

**Table 4:** **Comparison of mean concentrations of cytokines and chemokines in children with ASD (*n* = 22) and TD controls (*n* = 13)**.

	TD (*n* = 13)	ASD (*n* = 22)	*t*	*p*
**Cytokines**				
Migration inhibitory factor (ng/mL)	17.25 (5.35)	20.89 (11.30)	−1.09	0.29
Transforming growth factor beta 1 (pg/mL)	31530.81 (5746.58)	32228.70 (8917.55)	−0.25	0.80
**Chemokines**				
CCL2 (pg/mL)	141.78 (38.89)	182.21 (66.61)	−2.27	0.03*
CCL5 (pg/mL)	32396.60 (8061.28)	45273.38 (16620.41)	−3.07	0.004**
CXCL8 (pg/mL)	79.87 (18.20)	78.83 (33.00)	0.12	0.91
CXCL9 (pg/mL)	522.64 (212.87)	270.19 (100.92)	4.02	0.001**
CXCL10 (pg/mL)	1155.78 (493.21)	999.70 (415.49)	1.00	0.32

*Data are presented as means (SD)*.

*ASD, children with diagnosis of autism spectrum disorder; TD, typically developing children*.

*p < 0.05, **p < 0.01.

### Associations between Concentrations of Cytokines and Chemokines with Cognitive and Behavioral Measures in the ASD and TD Groups

Spearman’s rank-correlation analysis was performed to determine whether there were correlations between concentrations of cytokines and chemokines and the cognitive and behavioral domains among ASD and TD participants. To reduce the number of statistical comparisons and thereby avoid inflation of Type I error, composite *Z* scores were computed for each of the two cognitive (executive functioning and short-term memory) and three behavioral (social communication/interaction, repetitive/restricted behavior and inattention/hyperactivity) domains. First, individual *Z* scores for each cognitive or behavioral measure were computed based on the mean and *SD* obtained from normative sample statistics. The composite *Z* score of each cognitive and behavioral domain was then calculated by taking the average of the individual *Z* scores for the cognitive or behavioral measures tapping the corresponding domain.

Table 5 shows the specific associations between concentrations of cytokines and chemokines with cognitive and behavioral domains assessed among all participants. Generally, fewer significant associations were observed between concentrations of chemokines and the cognitive domains than between those concentrations and the behavioral domains. For the cognitive domains, significant associations were found between MIF, CXCL10, and EF scores, such that lower EF scores were significantly correlated with increased concentrations of MIF, *r* = −0.48, *p* = 0.003, and reduced concentrations of CXCL10, *r* = 0.49, *p* = 0.003. For the behavioral domains, significant associations were found between increased CCL5 and lower composite scores in all of the behavioral domains, namely social communication/interaction, *r* = −0.39, *p* = 0.02, repetitive/restricted behavior, *r* = −0.39, *p* = 0.02, and inattention/hyperactivity, *r* = −0.70, *p* < 0.001. Similarly, associations were also found between decreased concentrations of CXCL9 and poorer performance in all of the behavioral domains: social communication/interaction, *r* = 0.49, *p* = 0.003, repetitive/restricted behavior, *r* = 0.49, *p* = 0.003, except that a non-significant trend of association could only be detected in inattention/hyperactivity, *r* = 0.30, *p* = 0.08. Most of these associations between concentrations of cytokines and chemokines and the cognitive and behavioral domains showed moderate to large effect sizes, and the strongest association was found between increased CCL5 and lower composite scores for inattention/hyperactivity.

**Table 5:** **Whole-group association analysis of serum cytokines and chemokines with cognitive and behavioral domains in all participants enrolled in this study (*n* = 35) using Spearman’s rank correlations**.

	Migration inhibitory factor		Transforming growth factor beta 1		CCL2		CCL5		CXCL8		CXCL9		CXCL10	
	*r*_s_	*p*	*r*_s_	*p*	*r*_s_	*p*	*r*_s_	*p*	*r*_s_	*p*	*r*_s_	*p*	*r*_s_	*p*
Executive functioning	−0.48	0.003**	0.01	0.97	0.24	0.16	−0.16	0.36	0.10	0.58	0.26	0.13	0.49	0.003**
Short-term memory	−0.30	0.08	−0.10	0.56	−0.10	0.55	−0.19	0.27	−0.06	0.72	0.07	0.70	0.10	0.58
Social communication/interaction	−0.20	0.25	0.04	0.83	−0.12	0.51	−0.39	0.02*	−0.04	0.84	0.49	0.003**	0.19	0.27
Repetitive/restricted behavior	−0.10	0.55	0.00	0.99	−0.09	0.62	−0.39	0.02*	0.03	0.88	0.49	0.003**	0.18	0.30
Inattention/hyperactivity	−0.19	0.26	−0.17	0.32	−0.19	0.26	−0.70	<0.001**	−0.07	0.69	0.30	0.08	0.08	0.67

*p < 0.05, **p < 0.01.

### Associations between Concentrations of Cytokines and Chemokines with Cognitive and Behavioral Measures within the ASD Group

To determine whether there was specific relationship between measures of cytokines and chemokines and severity of cognitive and behavioral outcomes among children with ASD, a second correlation analysis was performed within the ASD group (as shown in Table 6). When the ASD group was analyzed alone, lower EF scores were found to be significantly correlated with increased MIF, *r* = −0.57, *p* = 0.005, and decreased CXCL10, *r* = 0.63, *p* = 0.002. In addition, we found a significant correlation between concentration of CXCL9 and STM scores, such that as CXCL9 increased, STM performance decreased, *r* = −0.59, *p* = 0.004. Regarding the behavioral domains, the only association observed was that between the concentration of CCL5 and composite scores of inattention/hyperactivity at a large effect size, such that as the concentration of CCL5 increased, more inattention and hyperactive symptoms occurred, *r* = −0.62, *p* = 0.002. However, no other association was observed between the composite scores of social communication/interaction, repetitive/restricted behavior and concentrations of cytokines and chemokines.

**Table 6:** **ASD subgroup association analysis of serum cytokines and chemokines with cognitive and behavioral domains in participants with ASD (*n* = 22) using Spearman’s rank correlations**.

	Migration inhibitory factor		Transforming growth factor beta 1		CCL2		CCL5		CXCL8		CXCL9		CXCL10	
	*r*_s_	*p*	*r*_s_	*p*	*r*_s_	*p*	*r*_s_	*p*	*r*_s_	*p*	*r*_s_	*p*	*r*_s_	*p*
Executive functioning	−0.57	0.005**	0.06	0.79	0.39	0.07	−0.14	0.53	0.26	0.24	−0.06	0.80	0.63	0.002**
Short-term memory	−0.28	0.21	−0.03	0.88	−0.15	0.51	−0.04	0.84	−0.03	0.88	−0.59	0.004**	−0.04	0.85
Social communication/interaction	−0.10	0.65	0.32	0.15	0.13	0.58	−0.13	0.58	−0.02	0.92	−0.05	0.83	0.28	0.20
Repetitive/restricted behavior	0.12	0.60	0.23	0.31	0.17	0.46	−0.05	0.82	0.16	0.47	−0.22	0.34	0.03	0.89
Inattention/hyperactivity	−0.37	0.09	−0.17	0.44	−0.16	0.49	−0.62	0.002**	−0.05	0.82	−0.36	0.10	−0.12	0.59

*p < 0.05, **p < 0.01.

### Comparison of Cognitive and Behavioral Measurements between Children with ASD-Only and Children with ASD + ADHD

To examine the associated deficits and immunological profiles in ASDs comorbid with other disorders, the children with ASD in the present study were further divided into two groups: those who were diagnosed with ASD-only and those with a comorbid diagnosis of ASD and ADHD (ASD + ADHD). As shown in Table 7, no significant differences in any of the cognitive and behavioral domains were observed between children with ASD-only and those with ASD + ADHD. However, differences between the two ASD groups in the EF score were observed such that children with ASD + ADHD tended to perform poorer than children with ASD-only in the EF domain, *p* = 0.07. Children with ASD + ADHD also tended to show lower performance in the STM domain and had more features in the inattention/hyperactivity domain than did children with ASD-only. However, no significant between-group difference was detected, possibly because of the relatively small sample size in each of the ASD subgroups.

**Table 7:** **Comparison of mean composite scores in cognitive and behavioral domains between children with ASD only (*n* = 13), children with comorbid diagnosis of ASDs and attention deficit hyperactivity disorder (*n* = 9), and TD controls (*n* = 13)**.

	TD (*n* = 13)	ASD		*F* or α*2*	*p*	*Post hoc* results
		ASD-only (*n* = 13)	ASD + ADHD (*n* = 9)			
Executive functioning	−0.30 (0.50)	−0.50 (0.59)	−1.02 (0.50)	5.06	0.01*	TD < ASD + ADHD
Short-term memory	−0.08 (0.70)	−0.61(0.67)	−0.94 (0.50)	4.96	0.01*	TD < ASD + ADHD
Social communication/interaction^a^	3.10 (0.37)	−0.29 (1.24)	−0.05 (1.21)	65.85	<0.001**	TD < ASD, ASD + ADHD
Repetitive/restricted behavior^a^	1.53 (0.21)	−1.08 (1.13)	−0.59 (0.97)	50.34	<0.001**	TD < ASD, ASD + ADHD
Inattention/hyperactivity^a^	0.22 (0.51)	−1.28 (1.20)	−1.75 (1.04)	18.89	<0.001**	TD < ASD, ASD + ADHD

*Data are presented as means (SD)*.

*ASD, children with diagnosis of autism spectrum disorder; ASD-only, children with diagnosis of autism spectrum disorder only; ASD + ADHD, children with comorbid diagnosis of autism spectrum disorder and attention deficit hyperactivity disorder; TD, typically developing children*.

*^a^Welch’s test and Games–Howell post hoc test were performed for variables violating the homogeneity of variances assumption of analysis of variance test*.

*p < 0.05, **p < 0.01.

### Comparison of Immunological Profiles among Children with ASD-Only and Those with ASD + ADHD

**Figure 1:**
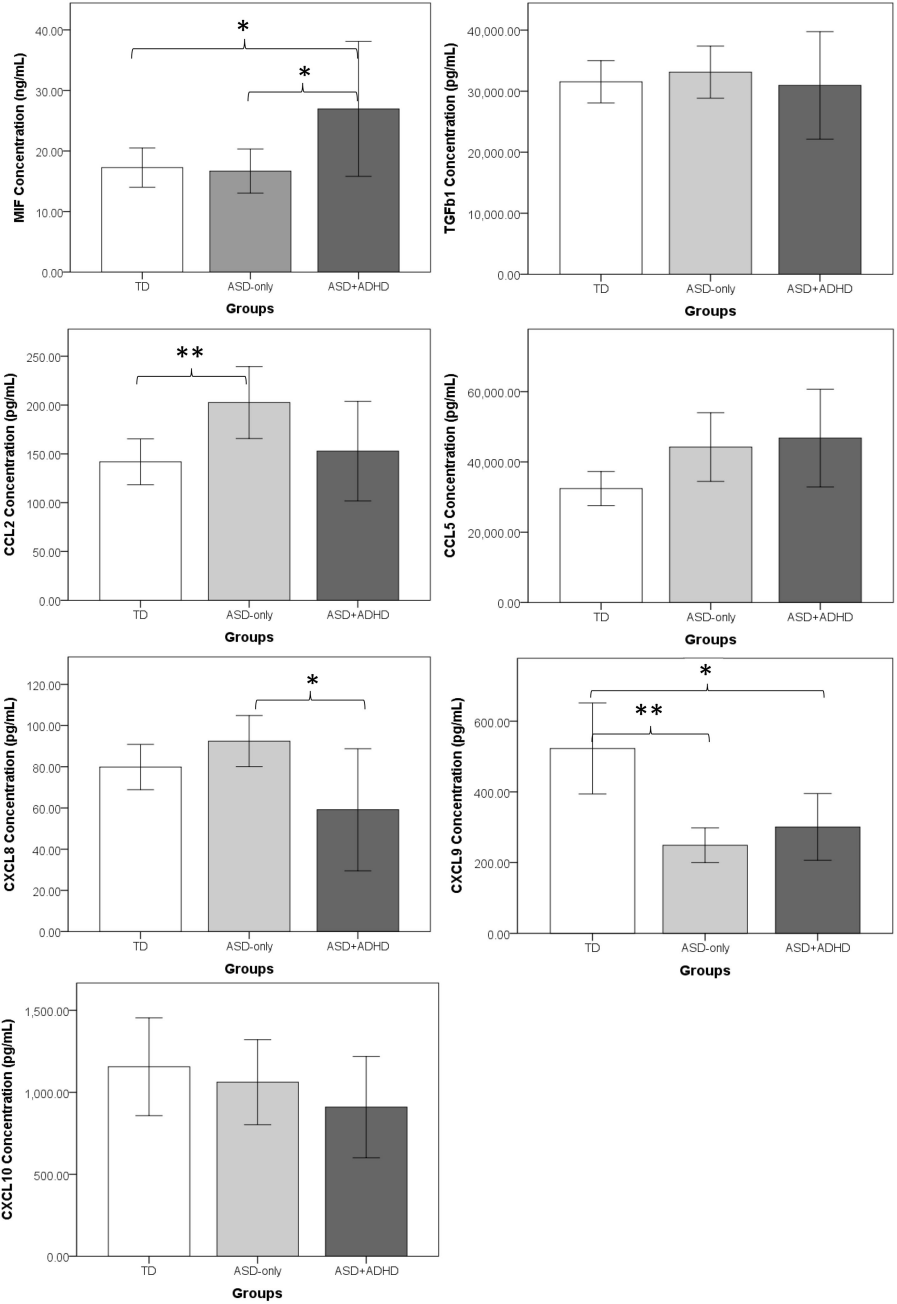
**Comparisons of mean concentrations of cytokines and chemokines between the ASD-only, ASD+ADHD, and TD groups**. The error bars represent a 95% confidence interval around the mean. TD, typically developing children; ASD-only, children with diagnosis of autism spectrum disorder; ASD + ADHD, children with comorbid diagnosis of ASD and attention deficit hyperactivity disorder (ADHD). *p < 0.05, **p < 0.01.

Two separate MANOVA tests were performed to compare, first, the concentrations of cytokines and, second, the concentrations of chemokines among ASD-only, ASD + ADHD and TD controls, followed by *post hoc* Tukey HSD tests to examine the pair-wise group differences in concentrations of cytokines and chemokines. Figure 1 shows the mean concentrations of cytokines and chemokines in each group. Results of MANOVA showed that there were significant between-group differences in both the profiles of cytokines, *F*(4, 62) = 2.90, *p* = 0.03, ${\text{η}}_{p}^{2}=0.16$, and the profiles of chemokines, *F*(10, 56) = 3.66, *p* = 0.001, ${\text{η}}_{p}^{2}=0.40$. Moreover, separate univariate ANOVAs on the dependent variables revealed that there were significant group differences in one cytokine [MIF: *F*(2, 32) = 4.35, *p* = 0.02, ${\text{η}}_{p}^{2}=0.21$], and four chemokines [CCL2: *F*(2, 32) = 4.32, *p* = 0.02, ${\text{η}}_{p}^{2}=0.21$; CCL5: *F*(2, 32) = 3.40, *p* = 0.05, ${\text{η}}_{p}^{2}=0.18$; CXCL8: *F*(2, 32) = 4.52, *p* = 0.02, ${\text{η}}_{p}^{2}=0.22$; CXCL9: *F*(2, 32) = 11.52, *p* < 0.001, ${\text{η}}_{p}^{2}=0.42$]. In contrast, there were no significant group differences with respect to the concentrations of TGFβ1, *F*(2, 32) = 0.23, *p* = 0.80, ${\text{η}}_{p}^{2}=0.01$, or CXCL10, *F*(2, 32) = 0.80, *p* = 0.46, ${\text{η}}_{p}^{2}=0.05$. Since the ASD-only group contained significantly more males than the TD and the ASD + ADHD groups, MANOVA tests were performed to examine whether there was gender difference in the cytokine and chemokine expressions within the ASD + ADHD and TD groups of children. Results showed that there was no significant gender difference in the profiles of cytokines or chemokines in the ASD + ADHD or TD groups, *p*s > 0.30. Moreover, results of separate univariate ANOVAs on the dependent variables also revealed that there was no significant group difference in any of the immunological measures between the males and females in the ASD + ADHD and TD groups, *p*s > 0.05. The results suggest that the females in the ASD + ADHD and TD groups exhibit similar cytokine and chemokine profiles as compared to their male counterparts and that the sex difference is not a confounding factor in the immunological measures between these groups.

#### Deviated Concentrations of Cytokines and Chemokines in ASD-Only Children

Regarding the deviated concentrations of cytokines and chemokines in ASD-only children, *post hoc* Tukey HSD tests showed that ASD-only children demonstrated significantly higher CCL2 concentrations, *p* = 0.02, and lower CXCL9 concentrations, *p* < 0.001, than did the TD controls. In contrast, there were no significant differences between the ASD-only children and TD controls in the concentrations of two cytokines (MIF: *p* = 0.99; TGFβ1: *p* = 0.87) or three other chemokines (CCL5: *p* = 0.10; CXCL8: *p* = 0.43; CXCL10: *p* = 0.86) (Figure 1).

#### Deviated Concentrations of Cytokines and Chemokines in ASD Children Comorbid with ADHD

*Post hoc* Tukey HSD test results showed that ASD + ADHD children demonstrated a dissimilar immunological profile compared with that of ASD-only children (Figure 1). With respect to cytokine concentration, the ASD + ADHD group demonstrated a significantly higher MIF concentration than did the TD group, *p* = 0.04, but no such deviated concentration was observed for the ASD-only group. With respect to chemokine concentration, a significantly elevated CCL2 pattern was observed for the ASD-only group, *p* = 0.02, whereas no significant elevation of CCL2 concentrations was found in the ASD + ADHD group compared to the TD group, *p* = 0.90. In addition, when comparing the immunological profiles of the ASD-only and ASD + ADHD group, the ASD + ADHD group demonstrated significantly higher MIF, *p* = 0.03, and lower CXCL8, *p* = 0.01, concentrations than did the ASD-only group. Nevertheless, it is noted that a significantly lower concentration of CXCL9 was observed in both the ASD-only, *p* < 0.001, and ASD + ADHD groups, *p* = 0.006, compared with the TD controls. Moreover, a trend of elevated CCL5 was observed in both the ASD-only, *p* = 0.10, and ASD + ADHD groups, *p* = 0.07, when compared to the concentrations measured for the TD controls, suggesting a small degree of similarity between the immunological profiles of the ASD-only and ASD + ADHD groups.

## Discussion

The present study examined cognitive function deficits and behavioral problems among a group of children with ASD who were aged 6–17 years and whether these deficits were associated with altered pro-inflammatory cytokine and chemokine profiles. The findings of the present study showed deviated concentrations of serum cytokines and chemokines, with elevated macrophage/monocytes CCL2 and levels of the Th2-related chemokine CCL5 and reduced levels of the T helper type 1 (Th1)-related chemokine CXCL9 in children with ASD compared to TD controls. Consistent results were obtained from the subgroup analyses involving only male participants from each group and after controlling for age differences, suggesting that neither gender nor age could account for differences in the immunological profiles between the two groups of children. In addition, the deviations in CCL5 and CXCL9 were linked to impairments in the behavioral domains, with associations observed between more behavioral problems measured in the three behavioral domains, including social communication/interaction, repetitive/restricted behavior, and inattention/hyperactivity, and increased levels of CCL5 and decreased CXCL9, such that impairments in the behavioral domains were more pronounced in individuals with deviations in the concentrations of these two chemokines. The present results are in line with those of previous studies showing an increase in plasma levels of CCL2 and CCL5 in individuals with ASD (1, 8). In what appears to be an important extension of previous studies, our findings are the first to show significantly reduced concentrations of CXCL9 in children with ASD compared to TD controls. The reduced levels of CXCL9 may be related to the slight decrease in the levels of the Th1-related chemokine CXCL10 in the ASD and ASD + ADHD groups, given that CXCL9 is a chemokine that is structurally and functionally related to CXCL10, which binds to a common inflammatory chemokine receptor, CXCR3, to coordinate inflammation in a variety of human diseases (9, 33). In fact, a positive correlation was found between levels of CXCL9 and CXCL10 in the present study, *r* = 0.35, *p* = 0.04, indicating that both of these chemokines may have important implications in the inflammatory mechanism in ASD, in that their levels of production show similar patterns. This finding may provide additional evidence of the involvement of CXCL9 in the pathophysiology of ASD for future research, given that CXCL9 has been relatively unexplored in previous studies. Furthermore, considering different cytokines/chemokines can be secreted by different T cell subsets, such as Th1, Th2, Th17, Th22, Th9, regulatory T cells, and follicular T helper cells; or leukocytes subsets including macrophages, dendritic cells, neutrophils, natural killers, and natural killer cells, it is reasonable to postulate that different cell number of the T cell subsets and leukocytes presented in ASD and the TD controls are responsible for the observed differences in cytokine/chemokine levels between the two groups of children.

However, in contrast to previous findings that have repeatedly indicated a significant reduction in TGFβ1 and a role for this reduction in the neuroinflammatory process of ASD (1, 4, 8–10), our results showed no difference in TGFβ1 between the TD and ASD groups. One possible reason could be related to the uniqueness of the genetic and environmental characteristics of different ethnic groups, varying immunological measures such as white blood cell counts and percentages of lymphocytes and granulocytes have been found to vary among different races (34, 35). For example, the association between decreased circulating concentrations of TGFβ1 and the severity of behavioral symptoms in ASD has been consistently reported in studies on Caucasians. In contrast, a study conducted on Japanese individuals with ASD did not demonstrate such an association between decreased serum levels of TGFβ1 and ASD symptoms (10). Another possible reason is the different biological samples analyzed. In most previous studies on peripheral levels of MIF and TGFβ1, samples were collected from plasma, whereas the samples used in the present study were collected from serum. It is still unknown whether the plasma level of cytokines directly reflects the serum level of the same cytokines (8, 10, 36–40). Nevertheless, given the relatively small sample size in the present study, further verification with a larger sample size is necessary before any firm conclusions can be drawn.

Furthermore, autism is a broad spectrum of disorders over which affected individuals demonstrate a broad range of symptoms in social and behavioral respects, with approximately 30–50% of ASD individuals showing prominent ADHD symptoms (15), and ASD children with or without ADHD have been found to exhibit distinct cognitive and behavioral profiles (15–19). For this reason, the present study also aimed to explore whether different immune mechanisms are active in these children. Interestingly, findings from the present study have demonstrated a robustly increased level of CCL2 in the ASD group but not in the ASD + ADHD group. Specifically, robust between-group differences were observed in the elevated concentration of CCL2 between our samples of ASD-only and TD controls. On one hand, these results agree with the majority of findings reported in the literature indicating that substantially higher concentrations of CCL2 are observed across different biological samples, including plasma, CSF, astrocytes in the anterior cingulate gyrus, and even tissue from the cerebellum, where elevated concentrations of CCL2 appear to be relatively stable across a broad age range (from newborn to middle-aged adults) and across various levels of intellectual functioning (with or without mental disabilities) among individuals with ASD (1, 6, 7, 14). Given the critical role of CCL2 in the proinflammatory process and its implication in neuroinflammation underlying the pathogenesis of certain CNS diseases, e.g., Alzheimer’s disease (41–45), it is postulated that the elevated CCL2 levels observed in the present study may indicate neuroinflammation in ASD. However, it should be noted that, in contrast to previous studies that showed associations between increased CCL2 and impairments in behavioral functions among individuals with ASD (6), the lack of between-group differences for the ASD + ADHD group and the absence of a significant association of CCL2 with any of the cognitive or behavioral measures considered in the present study warrants future work to delineate the role of CCL2 in the expression of autistic traits.

It is worth noting that although a non-significant trend of increased MIF and decreased CXCL10 was observed in children with ASD, significant associations were in fact detected between these deviations and poorer performance in the EF domain. Specifically, an increased MIF but reduced levels of the Th1-related chemokine CXCL10 were related to poorer executive performance. Furthermore, increased CXCL9 was associated with decreased short-term memory, and increased CCL5 was shown to be highly associated with more severe inattention/hyperactivity problems. Moreover, it is interesting to see that elevated MIF and decreased CXCL8 levels were found only in children with ASD comorbid with ADHD but not in children of the ASD-only group. Although the mechanism underlying the distinctive immunological profile of ASD and ASD + ADHD is unknown, this study provides important information indicating a potentially distinct cytokine/chemokine profile between different subtypes of autistic disorder. This notion is in agreement with previous studies that reported the opposite pattern of CCL5 concentrations between autistic individuals with and without FXS (14). In fact, our results seem to suggest that lumping ASD individuals with varying phenotypes into one group for analysis may in fact dilute the between-group difference relative to the control. Given the relaxed diagnostic criteria of the DSM-V that allows for the dual diagnosis of ASD and ADHD, it is worth further exploring the immunological difference between individuals with ASD-only and those with a co-diagnosis of ADHD in future studies. For example, it will be of interest to investigate the functional role of CCL5 in individuals with ADHD and to examine further whether CCR5 blockers will improve attention in animal models of ASD.

Although interesting associations that suggest the role of inflammatory chemokines and cytokines in the pathogenesis of ASD were observed in the present study, it should be noted that our findings of the association between concentrations of cytokines and chemokines and behavioral functions in ASD were not as strong as we hypothesized. A significant association was detected only between increased levels of the Th2-related chemokine CCL5 and more inattention/hyperactivity problems, which is in contrast with previous studies reporting a significant association between increased levels of CCL5 and greater impairments in social and communication skills in individuals with ASD (6). Nevertheless, the extent of all associations found in the present study had a larger effect size compared to that of the significant correlations detected by Ashwood et al. (6). Additionally, regarding the moderate degree of association between increased CXCL9 levels and deficits in short-term memory, the finding is a novel one in the neuro-immunological field of research on ASD. Although the underlying mechanism of aberrant serum levels of CXCL9 on the pathogenesis of ASD is not known, given its close association with CXCL10, it may also play a role in coordinating the inflammatory process in ASD (33, 46). Further investigation is needed to determine the link between decreased CXCL9 levels and better short-term memory performance in ASD patients. Moreover, our finding of the significant association between decreased serum levels of CXCL10 and poorer executive performance in ASD is in contrast to previous studies that showed an increased plasma level of CXCL10 was positively associated with disease progression and mental flexibility and inhibitory control in patients with Parkinson’s disease (47). Thus, future research may be necessary to clarify whether the link between immunological abnormalities and cognitive functioning differ between diseases even if they both exhibit a neuroinflammatory profile.

Taken together, our results are in agreement with findings in previous studies showing that cytokines are involved in neurodevelopment and neuronal function and that cytokine imbalance and dysregulation can have long term neurological consequences (48). Furthermore, the fact that cytokine expression is dependent on genetic and environmental influences (49) may help explain our findings that showed distinct immunological profiles in children with ASD of Chinese ethnicity from previous studies conducted in Western populations. Specifically, cytokines may represent a biomarker for genetic or environmental factors in autism. Of which, an individual may be genetically predisposed to mount an inappropriate immune response, either too robustly or too weakly, against an infectious or toxic substance which in turn could cause associated damage to the brain and other body systems, including the immune system, for the development of ASD. Moreover, an individual may lack appropriate genetic machinery to excrete and eliminate toxins; thereby causing their accumulation in tissue or organ and subsequently lead to an amplification of the toxin’s effects in a variety of body systems, including the brain and the immune system. Therefore, certain environmental challenge during a critical period of child development could have especially severe consequences, causing abnormal CNS function, altered immune phenotypes, and autism. Although findings from the present study may implicate potential areas where manipulation targeting individual cytokine–receptor interactions may impact on the behavior and immunity in ASD, whether this manipulation of the immune response represents a therapeutic strategy in the treatment of ASD remains uncertain. The reason being that cytokines/chemokines are a complex immune network and individuals with ASD of different ethnicity may have different genetic backgrounds and expose to different environmental factors, therefore therapeutic strategy targeting on one or two cytokines/chemokines may not be sufficient for providing a general treatment of ASD.

Overall, the present study provides preliminary findings regarding immunological dysfunction in the peripheral blood of children with ASD in a Chinese population. To the best of our knowledge, there are no previous studies such as this one in Chinese population and only few in other Asian cohorts. Moreover, what appears to be an important extension of previous studies is that in the present study, it was found that individuals with ASD that do and do not have ADHD symptoms exhibit distinct pro-inflammatory profiles; and that cognitive function deficits, repetitive stereotyped behavior and inattention/hyperactivity exacerbated as a function of altered plasma levels of chemokines and cytokines between the two different subtypes of autistic disorder. The findings from the present study bring together several diverse areas of research on autism and establish a link between immunological alteration and cognitive and behavioral impairments in autistic children with different ethnic backgrounds. However, the following should be noted when interpreting the data. One of the main limitations of our study was the small sample size used, which may have led to the low statistical power detected in comparing the levels of chemokine and cytokine production between groups and the behavioral and cognitive correlations with the levels of chemokines and cytokines, although a moderate to large effect size was found. Moreover, a relatively weak relation between the concentrations of chemokines and cytokines and behavioral performance was observed in our study, which was likely because the level of behavioral impairment in our sample was not as severe as expected but showed a narrow spread around the mean. Therefore, it was rather difficult to determine whether our observations reflect a true picture of the relationship between behavioral performance and immunological profiles in ASD among the general population. Further studies with a larger sample size will be required to verify the results before more conclusive interpretations can be made regarding the functional role of cytokines play in ASD. In addition, the number of detected cytokines/chemokines in the plasma is relatively low in the present study, and it may be argued that the correlations of cytokines/chemokines in different tissues are highly dependent on the types of studied inflammatory cytokines/chemokines. For example, circulating plasma levels of cytokines may have certain correlation with the local concentrations inside the brain or CSF such as TNF-alpha in ASD. But IL-6 was found to be present in CSF but not in plasma in ASD (50). Furthermore, the expression of some cytokines, e.g., IL-1, IL-2, TGF-β, and granulocyte-macrophage colony-stimulating factor, is actually controversial, and different studies have found various results in different tissues. Further investigations are therefore required to elucidate the correlations of cytokines in different sites such as plasma and the brain.

## Author Contributions

YH, WC, SS, TC, CW, MY, and AC contributed to the conceptualization of the study, subject recruitment, data collection and analysis, and drafting and revision of the manuscript. TC contributed to the analysis of blood samples.

## Conflict of Interest Statement

The authors declare that the research was conducted in the absence of any commercial or financial relationships that could be construed as a potential conflict of interest.
